# Photodynamic Effect of Riboflavin on Chitosan Coatings and the Application in Pork Preservation

**DOI:** 10.3390/molecules27041355

**Published:** 2022-02-17

**Authors:** Jiliu Pei, Shengyu Zhu, Yu Liu, Yukang Song, Feng Xue, Xiaohui Xiong, Chen Li

**Affiliations:** 1 College of Food Science and Light Industry, Nanjing Tech University, Nanjing 211816, China; jiliupei@njtech.edu.cn (J.P.); zhushengyu@njtech.edu.cn (S.Z.); liuyu0413@njtech.edu.cn (Y.L.); songyukang@njtech.edu.cn (Y.S.); sateam@njtech.edu.cn (X.X.); 2 School of Pharmacy, Nanjing University of Chinese Medicine, Nanjing 210023, China; xuefeng@njucm.edu.cn

**Keywords:** chitosan, riboflavin, UV irradiation, pork preservation, antibacterial activity

## Abstract

Riboflavin (RF) was considered to be possessed of photoactivity to generate reactive oxygen species (ROS) under ultraviolet (UV) light, which is thought to be a favorable antibacterial candidate. Herein, RF was incorporated into chitosan (CS) coatings and treated under UV with different exposure times (2, 4, and 6 h) to improve the physicochemical and antibacterial properties. The results showed that the light transmittance and antibacterial performance of chitosan coatings gradually increased with the extension of the UV irradiation time. The antibacterial ability of chitosan coatings correlated with the generation of ROS: ∙OH and H_2_O_2_, which achieved 1549.08 and 95.48 μg/g, respectively, after 6 h irradiation. Furthermore, the chitosan coatings with UV irradiation also reduced the pH value, total volatile basic nitrogen (TVB-N), ΔE, and total viable counts (TVC) and improved sensory attributes of pork. In conclusion, the UV irradiated chitosan coatings could be used as an environmentally friendly antimicrobial packaging material to effectively delay the spoilage of pork, maintain its sensory quality and prolong its shelf life.

## 1. Introduction

Pork is one of the most important sources of animal protein and fat in daily life, and the consumption and market demand of pork are huge, second only to beef. EU countries, which rank first in the per capita consumption of pork, consume approximately 35.5 kg of pork per capita each year. In 2020, Chinese people consumed 54.518 million tons of pork. The quality and appearance of pork on supermarket shelves are very important to consumers. However, meat products are prone to spoil because of their low oxidative stability, high content of unsaturated fatty acids, and susceptibility to microbial contamination, which can lead to changes in color, flavor, and texture [1]. In addition, improper operation or improper storage in the processing of meat products can easily cause them to be infected by pathogenic microorganisms, which will accelerate the degradation of protein and the rancidity of fat, thereby affecting the organoleptic properties of meat, leading to the loss of nutritional quality and even causing hidden public health hazards [2]. There are many methods that have been proposed to improve the quality and shelf life of meat products by preventing oxidation during processing and storage [3,4]. As consumers are increasingly concerned about the adverse effects of chemical preservatives, there is a growing interest in the use of natural biological macromolecules as packaging materials due to their rich resources and good biodegradability [5]. In the current stage, the application of edible antimicrobial films and coatings in food preservation is attracting widespread attention from the food industry and researchers. Edible coatings are of food-grade and can be applied in different ways, such as spraying, smearing, or soaking. The dried coating forms a transparent film on the surface of the food as a part of the final product, thereby blocking external oxygen and bacteria and killing bacteria on the surface of the food to a certain extent [6]. The coatings and films are usually made from polymers extracted from biomass, such as polysaccharides, proteins and lipids, which are the main materials for the production of biodegradable edible films [7]. Among them, CS has good physicochemical properties, biocompatibility, and antimicrobial and antifungal activities, thus it has become a very popular natural polymer material for food preservation and packaging in the food industry, and it has a wide range of application prospects [8].

CS usually exists in insect exoskeletons and crustaceans [9], and it is formed by deacetylation after the condensation of N-acetyl-D glucosamine and β (1–4) glycosidic bonds [5]. CS is a non-toxic natural polysaccharide with high biocompatibility and biodegradability [10]. It is colorless, odorless, and has excellent film-forming ability [11]. Moreover, CS can also carry a variety of functional substances to improve the synergistic effect of the entire preservative system, such as natural antioxidants and antimicrobial agents [5,12]. Furthermore, CS can be used to extend the shelf life of poultry meat through retarding the oxidative rancidity and microbial growth as well [13]. Therefore, it is considered an excellent raw material for making food coatings and biological preservatives to maintain the freshness and safety of food [14].

Riboflavin, commonly known as vitamin B_2_, is an essential water-soluble vitamin for human health and is widely found in dairy products, meat, and grains [15,16,17]. It has been reported to be one of the photosensitizers, which can be activated in certain wavelengths to produce reactive oxygen species with strong oxidation, so as to inactivate malignant cells and pathogenic microorganisms [18]. In addition, RF is generally considered to be safe because it can be excreted though the urine when the body over consumes, thus it can be added in many food products as a dietary supplement [19]. Furthermore, RF has shown the potential of blocking UV and visible light to inhibit or delay the photo-oxidation of food during the shelf life, and its transfer from the packaging matrix to the food during storage poses the least safety risk [20]. The unique properties and biological activities of RF molecules are directly related to its chemical structure, which creates great potential for making RF a mediator to prepare polymer functional materials. The critical structure that determines RF to belong to the flavin family is a tricyclic structure 7,8-dimethyl-10-alkylisoalloxazine. This essential fragment is responsible for the redox process, subsequent catalytic activity, UV absorption, and photosensitivity [21,22,23].

Many clinical studies have been conducted on the role of RF in combination with biopolymers in tissue engineering, transplantation, and corneal rejuvenation [24]. However, the research on the preparation of UV-induced CS-RF coatings is still insufficient. In this study, RF was incorporated as a photosensitizer into the CS matrix and irradiated by UV to prepare the CS-RF coatings to exert improved antibacterial properties. The coatings were first tested for their optical properties, in vitro bacteriostatic ability, and ROS generation. Furthermore, the coatings were applied to the preservation of fresh pork, and the TVB-N, pH, TVC, color, and sensory qualities of coated pork samples after 0, 2, 4, 6, and 8 days of storage were measured to comprehensively compare and evaluate the fresh-keeping ability of coatings, so as to provide a basis for expanding the application of CS-RF coatings in the food industry.

## 2. Results and Discussions

### 2.1. Optical Properties

**Figure 1:**
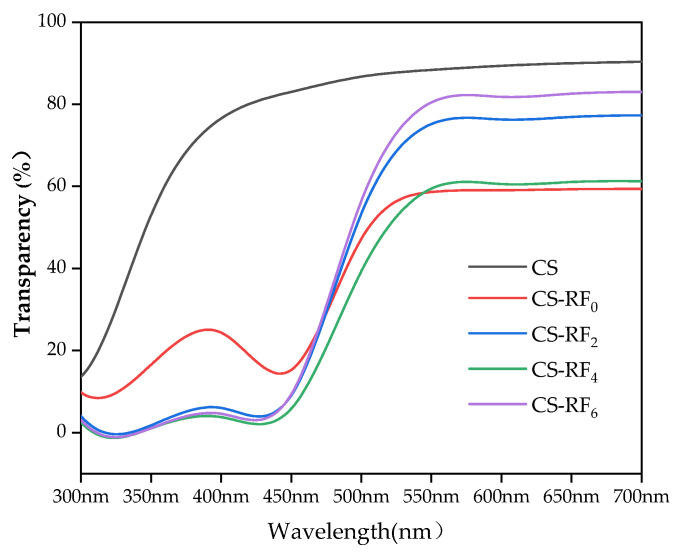
UV-vis transmission spectra of CS and CS-RF coatings (CS: chitosan coatings. CS-RF_0,2,4,6_: CS coatings incorporated with riboflavin by 0, 2, 4, 6 h of irradiation, respectively).

The light-barrier property of packaging material plays an important role in preventing light transmission (especially UV) because light can cause changes in food products, such as lipid oxidation, discoloration, nutrient losses, and odor [20]. The transmittance of UV and visible light through the coatings between 300–700 nm is shown in Figure 1. At 300–440 nm, the transmittance of CS solution added with RF was all less than 20%, which was significantly lower (*p* < 0.05) than that of pure CS, due to RF absorbing light from 200–500 nm [25]. Therefore, it can be seen that CS-RF coatings can help block UV rays. While in the range of 550–700 nm, the transmittance of the UV-irradiated coatings maintained at about 80% and gradually increased with the increase of the UV durations. Until 6 h of irradiation, the visible light transmittance of CS-RF coatings was similar to that of the CS coatings, which achieved the purpose of not changing its light transmittance as much as possible. This finding is important because transparency is critical in the application of coatings, especially when the coatings are used as a food covering or used to display the appearance of a packaged product. Ahmad et al. [26] reported that edible films with clear optical properties are more popular, with higher applicability and acceptability. Overall, light-induced CS-RF coatings can effectively inhibit UV light but has little effect on the transmission of visible light.

### 2.2. Antibacterial Activity

**Figure 2:**
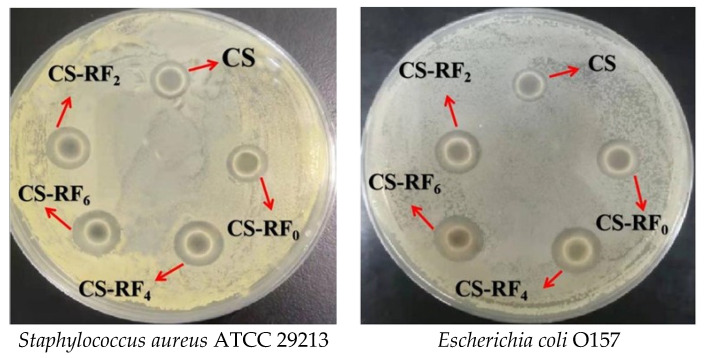
Antibacterial activity of CS and CS-RF coatings (CS: chitosan coatings. CS-RF_0,2,4,6_: CS coatings incorporated with riboflavin by 0, 2, 4, 6 h of irradiation, respectively).

As shown in Figure 2, both the CS and the CS-RF coatings have a significant inhibitory effect on *S. aureus* and *E. coli*. Part of the reason is that CS is a natural biopolymer, and many researchers have demonstrated that CS has good antibacterial activity against gram-positive bacteria, gram-negative bacteria, fungi, yeast, and other microorganisms [27,28]. Some researchers speculate that the antibacterial mechanism of CS may be due to the interaction of negatively charged microbial cell membranes with positively charged amine groups in CS, which changes the barrier properties of cell membranes, resulting in leakage of intracellular contents, and ultimately leading to cell death [27]. However, the antibacterial effect of pure CS was weak; the size of its inhibition zone against *E. coli* and *S. aureus* was 1.10 ± 0.36 mm and 2.27 ± 0.40 mm, respectively (Table 1). When RF was added, its antibacterial activity was slightly improved, which may be because of the antibacterial activity of RF itself. It has been reported that riboflavin-mediated 460 nm light-emitting diodes can inhibit *Listeria monocytogenes* [29] and *Salmonella* [30] both in the planktonic state and on the surface of food substrate. As the irradiation time increased, its antibacterial effect was significantly enhanced. After 6 h of light induction, the inhibition zone of the coatings on *E. coli* and *S. aureus* were 6.37 ± 0.15 mm and 7.61 ± 0.32 mm, respectively. The reason for the enhanced bacteriostasis may be due to the fact that RF is a photoinitiator widely used for free radical polymerization to prepare functional polymer materials, and it is usually used as an electron donor in two-component initiation systems [31,32]. For this reason, RF improved the structure of CS molecules to a certain extent and caused cross-linking between CS molecules. The photoinitiation function of RF in the radical polymerization of acrylamide has been extensively studied [33,34]. Besides, the enhanced antibacterial effect may be because RF is a type II photoinitiator, which reacts with electron acceptors or donors, or hydrogen donors in an excited state, to generate ROS [35,36]. ROS can cause severe oxidative stress, leading to DNA/RNA damage, lipid peroxidation, protein damage, enzyme inhibition, etc., leading to microbial cell death, which further enhances the antibacterial effect of the coatings [37].

### 2.3. ROS

Spectrometry was used to detect photochemical reactions. In the process of UV-irradiated CS coatings, the light-excited RF was decomposed into lumiflavin and lumichrome under certain illumination conditions. Therefore, the ROS produced by the intermediate can be detected after riboflavin is exposed to light, including ∙OH, H_2_O_2_, and ^1^O_2_, while the groups treated without light cannot detect the production of ROS [38,39]. The results are shown in Table 2. The generation of OH increased significantly, which for 6 h irradiation was three times of that for 2 h, and the production of H_2_O_2_ for 6 h was 1.6 times of that for 2 h, indicating that ∙OH and H_2_O_2_ were continuously produced during the process of 6 h of light induction. Zhang [40] fabricated nanofiber membranes that contained vitamin K_3_ as a photosensitizer; the ∙OH and H_2_O_2_ production of the membrane under one-hour D65 irradiation were 948 and 3292 μg/g, respectively. Compared to which, even though the ∙OH and H_2_O_2_ generated was not sufficient, the results suggested that the antibacterial effect correlated with the production of the ∙OH and H_2_O_2_. With the increase of irradiation time, the amount of the produced ^1^O_2_ increased, but the difference between 2 h and 6 h was not significant. The reason may be that the intramolecular electron transfer could compete with the intersystem crossing process as UV exposure prolonged, which lowers the ^1^O_2_ production [40].

### 2.4. pH

**Figure 3:**
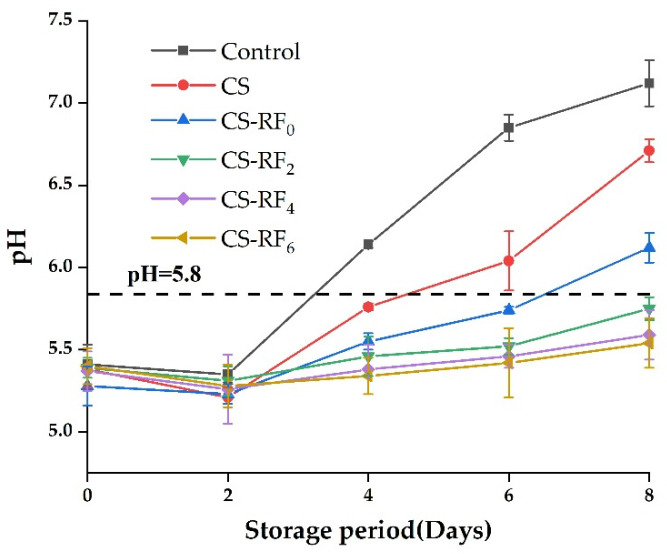
Changes of pH in coated pork during storage. (Control: pork samples without coatings. CS: pork samples with chitosan coatings. CS-RF_0,2,4,6_: pork samples with CS coatings incorporated with riboflavin by 0, 2, 4, 6 h of irradiation, respectively).

The pH value is one of the critical parameters that affect the balance of microorganisms in meat, and it shows the antibacterial effect of antibacterial agents [41]. The pH values of coated pork during eight days of refrigeration storage are shown in Figure 3. The pH value of the initial meat products was around 5.4–5.5, and all samples showed a trend of decrease first and then an increase during storage. This is owing to the anaerobic decomposition of pork due to the interruption of oxygen supply for glycolysis after slaughter, and the decomposition product was lactic acid, which lowered the pH value of pork temporarily [13]. In the subsequent storage process, the enzymes of glycogen anaerobic glycolysis were inhibited by lactic acid, and the protein in the pork samples was degraded into volatile basic nitrogen molecules through microbial activity and meat endogenous proteases. The alkaline amines (ammonia, primary amine, secondary amine, and tertiary amine) generated made the pH of the pork continue to increase [42]. However, the UV-induced CS-RF coatings had good antioxidant and antibacterial activities, inhibited protein degradation, produced less amines, and resulted in slower pH changes. Among them, the pork treated with the irradiated coatings made the pH lower than 5.8 on the eighth day, which was five days longer than that of the control group and three days longer than that with CS coatings. Huang [43] studied the grafting of caffeic acid onto the modified chitosan prepared by lipase, then applied it to the preservation of pork, and found that the pH did not change significantly within 17 days and remained at about 5.7 due to its strong antioxidant and antibacterial activities. This result was probably due to the UV-irradiated RF increasing the antibacterial and anti-oxidant properties of the coatings, thereby inhibiting the growth of microorganisms and delaying the spoilage of the meat.

### 2.5. TVB-N

**Figure 4:**
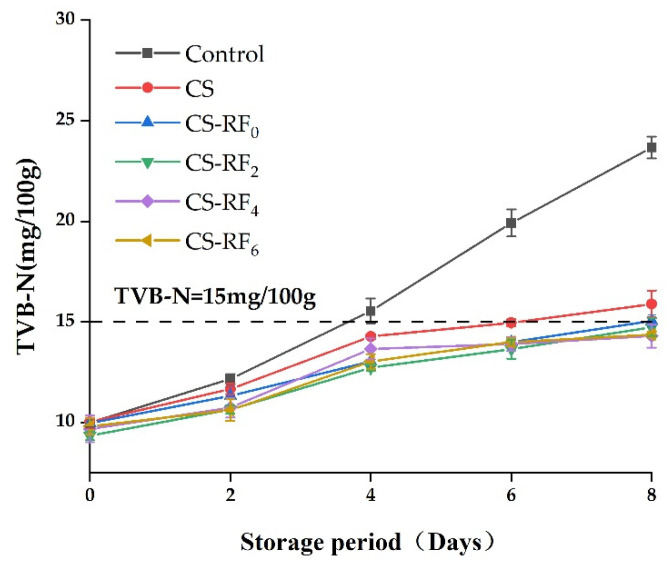
Changes of TVB-N in coated pork during storage. (Control: pork samples without coatings. CS: pork samples with chitosan coatings. CS-RF_0,2,4,6_: pork samples with CS coatings incorporated with riboflavin by 0, 2, 4, 6 h of irradiation, respectively).

The values of TVB-N are one of the most important freshness indicators for meat and meat products [44]. It can be seen from Figure 4 that the TVB-N of differently coated pork samples showed an upward trend during storage. This is because, in the course of storage, the increase of TVB-N was related to the degradation of non-protein nitrogenous compounds (such as nucleic acids) and protein components caused by exudation of proteolytic enzymes and the growth of microorganisms [45]. The limit of the national standard for food safety GB2707–2016 (TVB-N ≤ 15 mg/100 g) and the control group reached 15.5 mg/100 g on the fourth day, while the UV-irradiated groups did not exceed the standard until the end [46]. This may be due to the fact that CS coatings not only had higher antimicrobial activity, but also inhibited the lipid oxidation [47], and the addition of RF further enhanced the antibacterial performance of the coatings, thereby reducing the production of spoilage microorganisms, as well as slowing down the decomposition of protein. The UV durations had no significantly different effect on the TVB-N of coated pork; however, UV irradiation is beneficial for CS-RF coatings to further inhibit the TVB-N increase.

### 2.6. Color Characteristics

**Figure 5:**
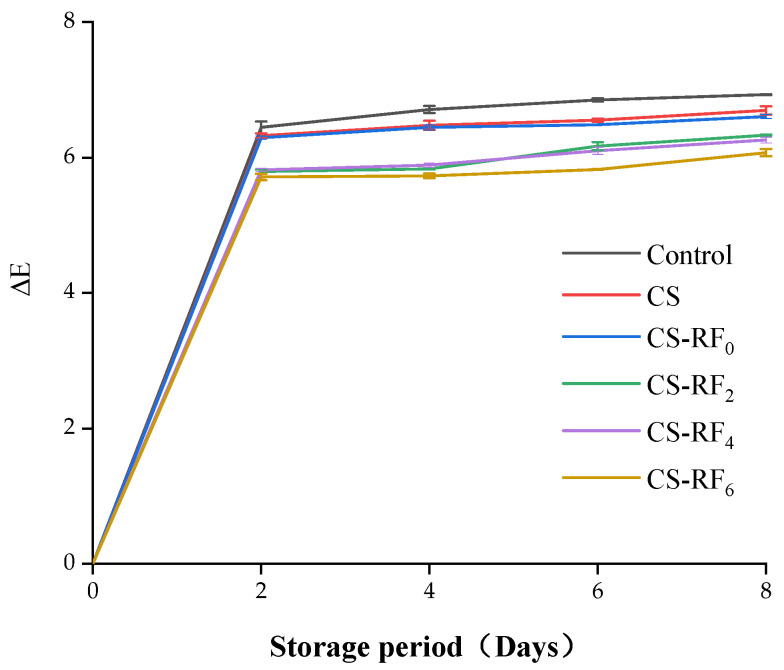
Color parameters of coated pork during storage. (Control: pork samples without coatings. CS: pork samples with chitosan coatings. CS-RF_0,2,4,6_: pork samples with CS coatings incorporated with riboflavin by 0, 2, 4, 6 h of irradiation, respectively).

Meat color is one of the important factors that consumers will consider when purchasing meat [48]. According to the survey, pork with high redness and low yellowness values is preferred by consumers because decreased redness and increased yellowness are usually related to the intensive degree of lipid oxidation and rancidity. The color changes of pork were quantified in terms of brightness (L*), redness (a*), and yellowness (b*) [49]. As shown in Figure 5, the total color difference (ΔE) of pork samples in different treatment groups was positively correlated with the storage time. The color of the pork is affected by various factors, such as pH value, oxidation, and microbial growth [50]. Therefore, reducing lipid oxidation and controlling the growth of microorganisms are beneficial to inhibit the rate of deterioration reaction and maintain the color of pork [51]. The results clearly demonstrated that compared with the control group, the ΔE of the pork in the coated groups was lower. The increased ΔE of the samples indicated that the deterioration degree of the samples was more obvious [52]. The coatings treated with UV irradiation showed better color stability to pork, which may be due to their better anti-oxidative, anti- ultraviolet, and antibacterial properties. On the eighth day, the ΔE of pork in the control group and the CS group reached 6.95 and 6.70, respectively, while the ΔE of the pork in CS-RF_6_ group was 6.07, which was a 12.7% and 9.4% reduction compared with that of the control and CS groups. It can be concluded that coating treatment can delay the deterioration of pork color, and the CS-RF_6_ coatings possessed the best effect.

### 2.7. TVC

**Figure 6:**
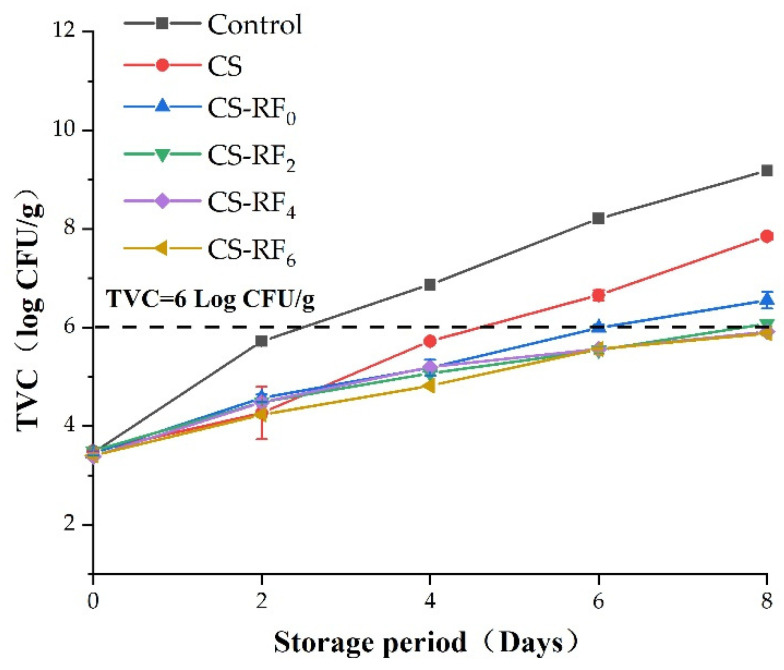
Changes of TVC in coated pork during storage. (Control: pork samples without coatings. CS: pork samples with chitosan coatings. CS-RF_0,2,4,6_: pork samples with CS coatings incorporated with riboflavin by 0, 2, 4, 6 h of irradiation, respectively).

In order to verify the inhibitory effect of the coatings on the growth of microorganisms in pork samples, the TVC was measured (Figure 6). The results showed that both CS coatings and UV irradiated CS-RF coatings had a significant impact on the number of microorganisms during the shelf life of the pork, and the storage time of different treatment groups was positively correlated with the TVC of pork. Compared with the control group, the growth rate of the colony of the pork in the UV irradiated treatment groups was significantly slower. On the eighth day of storage, the TVC of the control group and the CS group reached 9.26 log CFU/g and 7.89 log CFU/g, respectively, while the TVC of the CS-RF_6_ group was 5.86 log CFU/g, which was 99.96% and 99.06% lower than that of the control group and the CS group. In general, pork with a TVC of more than 6.0 log CFU/g is not allowed to be eaten [53]. Siripatrawan and Noipha [54] evaluated the effect of CS films on the number of microorganisms in pork sausages, which suggested an antimicrobial effect of the films against molds, yeasts, and lactic acid bacteria. Moreover, compared to CS-RF_0_ group, when CS-RF coatings were irradiated, it could better hinder the growth of microorganisms, delay the spoilage of meat products, and extend the storage period for at least two days.

### 2.8. Sensory Qualities

**Figure 7:**
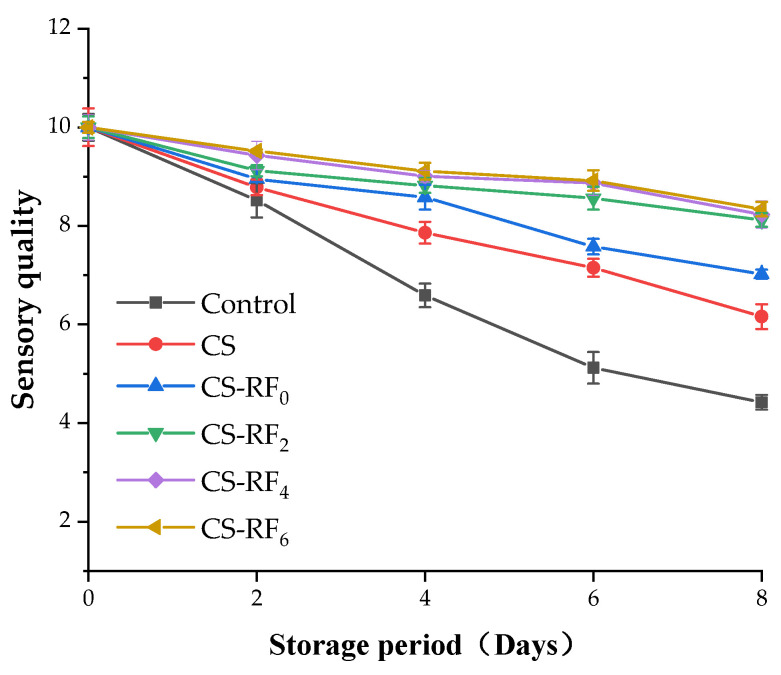
Sensory quality of coated pork during storage. (Control: pork samples without coatings. CS: pork samples with chitosan coatings. CS-RF_0,2,4,6_: pork samples with CS coatings incorporated with riboflavin by 0, 2, 4, 6 h of irradiation, respectively).

In daily life, people generally determine whether meat products are fresh by observing, smelling, and touching for sensory evaluation, and thus it is an important indicator of meat quality [55]. The sensory characteristics (odor, color, texture, and overall acceptability) of pork samples were scored on 0, 2, 4, 6, and 8 days, and the total score is shown in Figure 7. The sensory evaluation results were consistent with the experimental analysis results, and the sensory scores of pork samples in different treatment groups negatively correlated with storage time. Compared with the control group, the sensory quality of coated pork was significantly improved. Changes in sensory scores have been reported to correlate with microbial growth [56], and thus, the slow deterioration in the sensory qualities of treated pork may be due to the coatings inhibiting the oxidation and microbial growth, delayed color changes, and rancidity of pork samples. On the eighth day of storage, the sensory scores of the control group and the CS group pork reached 4.92 and 6.14, respectively, while the sensory score of CS-RF_6_ group was 8.32, which was 69.1% and 35.5% higher than that of control group and CS group. The sensory score changes indicated that the coating delayed the deterioration of the sensory quality of pork and had a better anti-corrosion effect. Compared with the CS coatings, the CS-RF coating was more effective in delaying the deterioration of the organoleptic quality of pork. Moreover, better sensory evaluation was discovered in the CS-RF_2_ group than the CS-RF_0_ group, while the sensory scores in UV-irradiated groups were not significantly different.

## 3. Materials and Methods

### 3.1. Materials

Chitosan (degree of deacetylation 90%), sodium hydroxide, and ammonium molybdate tetrahydrate were purchased from Yuanye Biotech Co., Ltd. (Shanghai, China). Riboflavin (purity 98–102%) and potassium iodide were obtained from Sangon Biotech Co., Ltd. (Shanghai, China). Glacial acetic acid (purity > 99.5%) was purchased from Yonghua Chemical Co., Ltd. (Shanghai, China). Glycerol and sodium chloride were both purchased from Xilong Science Co., Ltd. (Shantou, China). *p*-Nitroso dimethylaniline (*p*-NDA) was purchased from TCI Chemical Industry Development Co., Ltd. (Shanghai, China). Phosphate buffer solution, potassium hydrogen phthalate, and L-histidine were obtained from Aladdin Biochemical Technology Co., Ltd. (Shanghai, China). LB medium and agar powder were purchased from Hopebiol Biotech Co., Ltd. (Qingdao, China). *Escherichia coli* O157 and *Staphylococcus aureus* (ATCC29213) were both obtained from China General Microbiology Culture Collection Center (Beijing, China).

### 3.2. Preparation of CS-RF Coatings

Firstly, CS powder was dissolved into acetic acid (1% *v*/*v*) to prepare CS solutions (2% *w*/*v*), then 0.28 g/g CS amount of glycerin was added as a plasticizer. A magnetic stirrer (HJ-4, Jintan Baita Xinbao Instrument Factory, Changzhou, China) was used to mix continuously for 4 h at 25 °C until all the CS was dissolved [57]. Secondly, an appropriate amount of RF was added and stirred on a magnetic stirrer in the dark to obtain a homogeneous mixture. A 20 mL resulted mixed solution was cast into a disposable flat plate, then it was placed 10 cm away from UV light (15 W, F15T8/GL, Haichao, China) and irradiated for 0, 2, 4, and 6 h, respectively [7], which were marked as CS, CS-RF_0_, CS-RF_2_, CS-RF_4_, and CS-RF_6_. The prepared coatings were stored away from light in a glass desiccator with constant temperature and pressure (25 °C, 101.325 kPa).

### 3.3. Determination of Light Transmittance of CS-RF Coatings

A multifunctional microplate reader (Infinite E Plex, Shaanxi Always Biotech Co., Ltd., Xi’an, China) was used to measure the UV-visible transmission spectrum of the coatings [58]. The light transmittance of the coatings was measured in the wavelength range of 300–700 nm.

Transparency = 10^−A^ (1)

### 3.4. In Vitro Study on Antibacterial Activity

The Oxford cup method [59] was used to evaluate the antibacterial activity of the coatings against *E. coli* and *S. aureus*. Briefly, LB agar plates were prepared in glass Petri dishes, and 100 μL of S. aureus suspension (approximately 10^8^ CFU/mL) was evenly spread onto the LB agar plates. An amount of 100 μL of the coatings was added in the Oxford cup (inner diameter 6 nm, outer diameter 8 nm, height 10 nm) (Crisp Biotechnology Co., Ltd., Shanghai, China) and incubated in a 37 °C incubator for 24 h. The antibacterial activity of the coatings against *E. coli* and *S. aureus* was demonstrated by measuring the size of the inhibition zone [60].

### 3.5. Measurement of Hydroxyl Radicals

The production of hydroxyl radicals was quantitatively measured by instantly quenching the formed radicals with *p*-Nitroso dimethylaniline (*p*-NDA), a radical scavenger [61,62]. Coatings of different treatments were mixed with *p*-NDA (50 μM) solution in a volume ratio of 1:1. The decomposition of *p*-NDA was measured photometrically by referring to the absorption intensities at 440 nm. The production of ∙OH by the film solution can be quantitatively calculated according to the specific stoichiometry between ∙OH and *p*-NDA [62].

### 3.6. Measurement of Singlet Oxygen

For the singlet oxygen production test on the film solution, *p*-NDA (50 μM) and L-histidine (0.01 M) were dissolved in phosphate buffer solution (0.01 M, pH = 7.35) [40]. The production of singlet oxygen by the UV irradiation was obtained by the *p*-NDA consumption difference between L-histidine-added and L-histidine-free systems. The generation of singlet oxygen was reflected by the change of OD value [63].

### 3.7. Measurement of Hydrogen Peroxide

The production of hydrogen peroxide was detected by an indirect quantification method according to the previous study [64]. Potassium iodide (66 g/L), sodium hydroxide (2 g/L), and ammonium molybdate tetrahydrate (0.2 g/L) were dissolved in water to obtain reagent A. Potassium hydrogen phthalate (20 g/L) was dissolved in water to obtain reagent B. An amount of 1 mL of the coatings was extracted and mixed with 1 mL each of reagent A and reagent B, and the mixture was vigorously vortexed for 10 s to mix homogeneously. The mixture was subsequently placed in a dark environment for 5 min to react sufficiently. The concentration of the formed hydrogen peroxide in the coatings can be determined quantitatively according to the absorbance at 351 nm by using a UV-vis spectroscopy.

### 3.8. Microbiological Analysis in Pork Packaging

The quality characteristics of coated fresh pork was investigated according to previous study [65]. Fresh pork without fat and skin was sliced into 2 × 2 × 1 cm chunks and completely soaked in the different treated coatings for 30 s, then taken out and drained, placed in a disposable petri dish, sealed, and stored in a 4 °C refrigerator. The pH value of meat was determined according to Saricaoglu and Turhan [66] with slight modification. TVC was determined by the spread plate method through counting the microorganisms growing in LB medium. The sensory evaluation was carried out according to a slight modification of the method published by Chen et al. [67]. The TVB-N content of meat samples during storage was determined according to the Chinese National Food safety standard methods GB 5009.228–2016. Color parameters of the films were determined using a colorimeter (CR-300, Konica Minolta, Wayne, NJ, USA) [68]. The results were presented by L* (luminosity), a* (red, green), and b* (yellow, blue). With fresh pork as the control, the calculation formula of the total color difference ΔE is as follows:

∆E = [(L* − L)^2^ + (a* − a)^2^ + (b* − b)^2^]^1/2^ (2)

where L*, a*, and b* denote the data of different treatment groups, and L, a, and b denote data of the blank group on day 0. The higher the ΔE, the more obvious the deterioration of the samples.

### 3.9. Statistical Analysis

Analysis of variance (ANOVA) followed by a Tukey-b test were used when comparing more than two data sets, after confirming the homogeneity of variances by the Levene test using IBM SPSS Statistics software v.26. All data have been represented as the average ± standard deviation. Significant differences (*p* ≤ 0.05) are denoted by showing the data provided in tables with different letters.

## 4. Conclusions

This study verified the in vitro antibacterial effect of UV-irradiated CS-RF coatings and its potential for fresh pork preservation. UV-vis transmission spectra showed that the UV-induced CS-RF coatings exhibited good UV blocking ability and sufficient light transmittance. Moreover, the CS-RF coatings could generate ROS under UV irradiation, and the antibacterial effect against *Staphylococcus aureus* and *Escherichia coli* was enhanced with the prolongation of UV exposure time. The fresh pork preservation experiment proved the advantages of UV-induced CS-RF coatings in extending the shelf life. However, the UV durations suggested no significant difference in the preservation effect of fresh pork, except for ΔE. Therefore, more studies should be carried out in illustrating the effect of UV durations on the preservation of fresh products. In summary, the UV-induced CS-RF coating has a remarkable antibacterial property and has great potential as a biodegradable packaging material for extending the shelf life of chilled fresh products.

## Figures and Tables

**Table 1:** Antibacterial activity of CS and CS-RF coatings.

Samples	*S. aureus* (mm)	*E. coli* (mm)
CS	2.27 ± 0.40 ^a^	1.10 ± 0.36 ^a^
CS-RF_0_	3.03 ± 0.20 ^b^	1.97 ± 0.21 ^b^
CS-RF_2_	5.53 ± 0.49 ^c^	4.33 ± 0.31 ^c^
CS-RF_4_	6.80 ± 0.26 ^d^	5.73 ± 0.25 ^d^
CS-RF_6_	7.61 ± 0.32 ^e^	6.37 ± 0.15 ^e^

CS: chitosan coatings. CS-RF_0,2,4,6_: CS coatings incorporated with riboflavin by 0, 2, 4, 6 h of irradiation, respectively. Different letters within a column represent significant difference (*p* < 0.05).

**Table 2:** Generation of ROS in coatings.

Samples	∙OH (μg/g)	H_2_O_2_ (μg/g)	^1^O_2_
CS	-	-	-
CS-RF_0_	-	-	-
CS-RF_2_	465.48 ± 14.60 ^a^	58.57 ± 3.72 ^a^	0.17 ± 0.03 ^a^
CS-RF_4_	549.33 ± 22.65 ^b^	69.88 ± 2.51 ^b^	0.44 ± 0.08 ^b^
CS-RF_6_	1549.08 ± 129.41 ^c^	95.48 ± 1.69 ^c^	0.27 ± 0.06 ^a^

CS: chitosan coatings. CS-RF_0,2,4,6_: CS coatings incorporated with riboflavin by 0, 2, 4, 6 h of irradiation, respectively. Different letters within a column represent significant difference (*p* < 0.05).

## Data Availability

The data presented in this study are available on request from the corresponding author.

## Sample Availability

Samples of the compounds CS, CS-RF_0_, CS-RF_2_, CS-RF_4_, CS-RF_6_ coatings are available from the authors.
